# Clinical characteristics, prognosis, and surgical outcomes of patients with non-HBV and non-HCV related hepatocellular carcinoma: three-decade observational study

**DOI:** 10.1186/s12876-023-02833-0

**Published:** 2023-06-08

**Authors:** Koya Yasukawa, Akira Shimizu, Koji Kubota, Tsuyoshi Notake, Kiyotaka Hosoda, Hikaru Hayashi, Yuji Soejima

**Affiliations:** Division of Gastroenterological, Department of Surgery, Hepato-Biliary-Pancreatic, Transplantation and Pediatric Surgery, Shinshu University School of Medicine, Asahi 3-1-1, Matsumoto, 390-8621 Japan

**Keywords:** Liver, Surgical Nutrition and Metabolism, Viral hepatitis, Hepatectomy

## Abstract

**Background:**

The incidence of non-hepatitis B virus, non-hepatitis C virus hepatocellular carcinoma (non-B non-C-HCC) is increasing worldwide. We assessed the clinical characteristics and surgical outcomes of non-B non-C-HCC, versus hepatitis B (HBV-HCC) and hepatitis C (HCV-HCC).

**Methods:**

Etiologies, fibrosis stages, and survival outcomes were analyzed of 789 consecutive patients who underwent surgery from 1990 to 2020 (HBV-HCC, *n* = 149; HCV-HCC, *n* = 424; non-B non-C-HCC, *n* = 216).

**Results:**

The incidence of hypertension and diabetes mellitus was significantly higher in patients with NON-B NON-C-HCC than in those with HBV-HCC and HCV-HCC. Significantly more advanced tumor stages were observed in patients with non-B non-C-HCC; however, better liver function and lower fibrosis stages were observed. Patients with non-B non-C-HCC had significantly worse 5-year overall survival than patients with HBV-HCC; overall survival was comparable between patients with non-B non-C-HCC and HCV-HCC. Patients with HCV-HCC had significantly worse 5-year recurrence-free survival than patients with HBV-HCC and non-B non-C-HCC. In patients with non-B non-C-HCC, overall survival was comparable among three periods (1990–2000, 2001–2010, and 2011–2020) despite significant improvement in patients with HBV-HCC and HCV-HCC.

**Conclusion:**

The prognosis of non-B non-C-HCC was similar to that of HBV-HCC and HCV-HCC regardless of tumor progression at surgery. Patients with hypertension, diabetes mellitus, and dyslipidemia require careful systematic follow-up and treatment.

## Introduction

Liver cancer continues to be a pressing global health issue, with an anticipated incidence of over one million cases by 2025. Hepatocellular carcinoma (HCC) represents the predominant form of liver cancer and comprises approximately 90% of all cases [1]. HCC can be classified into three groups according to the background liver disease: hepatitis B virus-induced HCC (HBV-HCC), hepatitis C virus-induced HCC (HCV-HCC), and non-HBV, non-HCV HCC (non-B non-C-HCC). The incidence of HBV and HCV hepatitis as a cause of HCC has been decreasing because of treatment with nucleoside or nucleotide analogues, interferons, and direct-acting antivirals. By contrast, the number of patients with non-B non-C-HCC [negative for both serum hepatitis B surface antigen (HBsAg) and anti-HCV antibody (HCV-Ab)] is increasing each year [2–4]. However, the clinical characteristics and surgical outcomes of patients with non-B non-C-HCC who undergo liver resection remain controversial.

The background of liver damage as a cause of HCC in patients with non-B non-C-HCC widely varies and includes alcoholic liver disease, non-alcoholic fatty liver disease, non-alcoholic steatohepatitis (NASH), autoimmune hepatitis, and other cryptogenic causes. Numerous researchers have reported the surgical outcomes of non-B non-C-HCC or metabolic HCC [2–8], and almost all found that the long-term outcomes of non-B non-C-HCC were better than or comparable to those of other etiologies. However, Hsu et al. [9] reported worse outcomes of non-B non-C-HCC because of late diagnosis. Kokudo et al. [10] noted that these discrepancies in the literature may be due to differences in background liver disease as well as the etiologies and treatment strategies of the control groups. Therefore, further research is needed to accurately define and reassess the clinical characteristics of non-B non-C-HCC.

The aim of this study was to investigate the clinical characteristics and surgical outcomes of non-B non-C-HCC, focusing on its differences from HBV-HCC and HCV-HCC.

## Methods

### Patients

In total, 1066 consecutive patients who underwent surgical resection of primary HCC at the Department of Surgery, Shinshu University Hospital from December 1990 to June 2020 were identified in a single-institution database. Of these patients, we excluded those who underwent non-first hepatectomy (*n* = 236) and non-curative resection (*n* = 34). Patients who were seropositive for both HBsAg and HCV-Ab (*n* = 7) were also excluded from in this study. Finally, 789 patients were included in this study and classified by background liver disease as follows: seropositive for HBsAg (HBV group, *n* = 149), seropositive for HCV-Ab (HCV group, *n* = 424), and seronegative for both HBsAg and HCV-Ab (NBNC group, *n* = 216). The pathological findings were prospectively documented in accordance with the Japanese standardized reporting format for liver cancers, and liver cirrhosis, microscopic vascular invasion, and intrahepatic metastasis were relabeled based on the American Joint Committee on Cancer staging system, 7th edition [11].

### Ethics approval and consent to participate

The study was approved by Ethics committee of Shinshu University Hospital (approval No.2022–5456). Informed consent was obtained from all study participants and the study was carried out in accordance with relevant guidelines and Declaration of Helsinki. However, the study did not include individuals below the age of 16 who are undergoing medical treatment, and it was not mandatory to obtain the consent of a parent or an equivalent legal guardian.

### Criteria for liver resection

Since 1990, all liver resections in our institution have been conducted based on the Makuuchi criteria [12]; this was described in detail in our previous report [13]. Briefly, in patients without ascites and with a normal serum bilirubin concentration, two-thirds of the nontumorous liver parenchyma can be removed in patients with an indocyanine green retention rate at 15 min (ICGR15) of < 10%, one-third of the liver parenchyma can be resected in patients with an ICGR15 of 10% to 19%, and Couinaud’s segmentectomy is indicated for patients with an ICGR15 of 20% to 29%. Basically, liver resection for primary HCC at our institution is carried out by anatomic resection; however, limited resection is indicated in patients with an ICGR15 of > 30% [12, 14, 15].

### Postoperative follow-up

After discharge, the patients were followed up every 3 months in our outpatient clinic by ultrasonographic examination and measurement of serum tumor markers such as alpha-fetoprotein (AFP) and des-gamma-carboxy prothrombin (DCP). Computed tomography, magnetic resonance imaging, or gadolinium ethoxybenzyl diethylenetriamine pentaacetic acid-enhanced magnetic resonance imaging was performed every 6 months or as necessary. Recurrence was detected and diagnosed by imaging findings.

Patients with HCC recurrence underwent repeat hepatectomy if their liver function was sufficient for liver resection. If not, medical management such as radiofrequency ablation, percutaneous ethanol injection therapy, transcatheter arterial embolization, or molecular targeted therapies was performed.

### Definitions

Excessive alcohol consumption was defined as alcohol intake of > 80 g/day [16]. Post-hepatectomy liver failure was diagnosed and graded according to the criteria of the International Study Group of Liver Surgery [17, 18]. A near or distant site of recurrence was defined according to the Couinaud classification. For example, recurrence in the same segment was classified as near the resection site. We defined operative mortality as intraoperative death, death within 90 days after the operation, and in-hospital death. Postoperative complications were diagnosed and graded based on the Clavien–Dindo classification [19].

Our institution aggressively performs anatomical resection according to Makuuchi’s criteria [20]. Anatomical resection was defined as complete removal of the tumor together with the portal veins bearing the tumor and the corresponding hepatic territory; namely, one Couinaud segment or a combination of adjacent territories of the subsegmental portal venous branches smaller than one Couinaud segment (which was identified by dye injection into the tumor-bearing portal vein branches) or Glissonean pedicle transection. Non-anatomic resection was defined as so-called partial resection not included in the above definition. Treatment of multiple HCCs was classified as non-anatomic resection, even if one partial resection was performed.

### Statistical analysis

Continuous variables were compared with the Mann–Whitney *U* test, and categorical variables were compared with the χ^2^ test or Fisher’s exact test. Overall survival (OS) and recurrence-free survival (RFS) were analyzed by the log-rank test and plotted by the Kaplan–Meier method. OS was analyzed from the date of surgical resection to the date of death of all causes, and RFS was defined as the duration from the date of initial diagnosis to the date of recurrence or death of any cause. We used variables to estimate the hazard ratio (HR) and 95% confidence interval (CI). Multivariate analyses were performed by forward selection of covariates that were identified as significant by univariate analysis with a cutoff *P* value of 0.05, after elimination of possible confounders. *P* values of < 0.05 were considered statistically significant. Transitions of patients’ background characteristics were analyzed using the Jonckheere–Terpstra test. Statistical analyses were performed using JMP® 16 (SAS Institute Inc., Cary, NC, USA).

## Results

### Patient characteristics

**Fig. 1:**
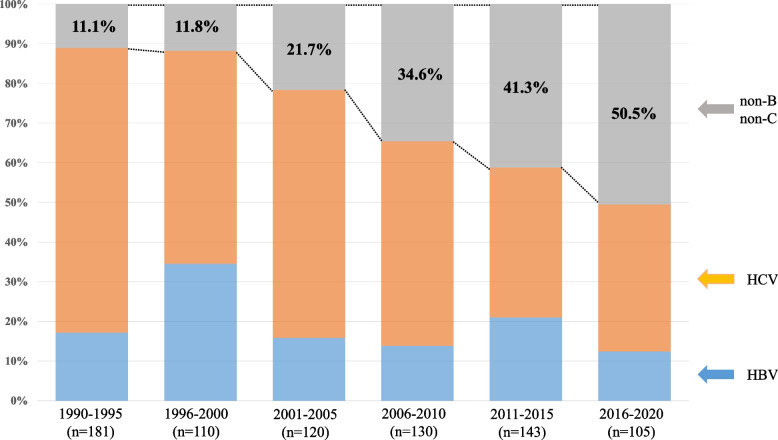
Changes in number of patients who underwent resection according to time period. HBV, hepatitis B virus; HCV, hepatitis C virus; non-B non-C, non-HBV, non-HCV

Changes in the patients’ background are shown in Fig. 1. The number of patients with non-B non-C-HCC increased over time (1990–1995: 11.1% vs. 2016–2020: 50.5%, *P* < 0.001), whereas the numbers of patients with HBV-HCC and HCV-HCC decreased (HBV, 1990–1995: 17.1% vs. 2016–2020: 12.4%, *P* = 0.071; HCV, 1990–1995: 71.8% vs. 2016–2020: 37.1%, *P* < 0.001). The differences in clinical characteristics, pathological findings, and surgical short-term outcomes among patients with HBV-HCC, HCV-HCC, and non-B non-C-HCC are summarized in Table 1.

**Table 1:** Clinicopathological characteristics and surgical short-term outcomes according to background of HCC

Variable	HBV-HCC (*n* = 149)	HCV-HCC (*n* = 424)	non-B non-C-HCC (*n* = 216)	*P* value	
				HBV vs. non-B non-C	HCV vs. non-B non-C
Host-related factors					
Age, years	60 (16–83)	69 (39–85)	71 (33–89)	< 0.001	0.032
Sex, male/female	114/35	306/118	173/43	0.413	0.027
BMI, kg/m^2^	23 (16–35)	22 (16–35)	23 (13–45)	0.354	< 0.001
HT	30 (22.7)	122 (36.3)	56 (49.1)	< 0.001	0.016
DM	17 (11.4)	88 (20.8)	102 (47.2)	< 0.001	< 0.001
Dyslipidemia	12 (9.1)	57 (16.9)	34 (15.7)	0.192	0.781
Heavy drinking	68 (45.6)	164 (38.7)	134 (62.0)	0.002	< 0.001
Smoking	81 (54.4)	211 (49.8)	139 (64.4)	0.066	< 0.001
Serum Alb, g/dl	4.0 (2.4–4.9)	3.8 (2.2–5.1)	3.9 (2.8–5.2)	0.875	< 0.001
Serum AST, IU/L	37 (16–178)	46 (6–293)	33 (10–142)	0.064	< 0.001
Serum ALT, IU/L	39 (9–134)	43 (5–420)	34 (8–215)	0.016	< 0.001
Serum T.bil, mg/dl	0.9 (0.4–1.9)	0.8 (0.2–2.7)	0.8 (0.3–2.8)	0.048	0.565
Serum Cre, mg/dl	0.74 (0.4–10.4)	0.75 (0.1–9.88)	0.8 (0.4–11.6)	0.015	0.005
Prothrombin time, %	82 (26–116)	88 (46–125)	89 (32–130)	< 0.001	0.222
Platelet count, 10^4^/ml	13.9 (3.9–50.4)	12.1 (3.0–53.1)	16.2 (4.1–41.4)	0.001	< 0.001
Fib 4 index	2.5 (0.3–14.3)	3.9 (0.9–16.4)	2.6 (0.4–14.5)	0.975	< 0.001
ICGR15, %	13 (2–47)	18 (4–90)	12 (3–89)	0.773	< 0.001
AFP, ng/ml	22 (0.7–999,999)	24 (0.3–184,000)	89 (0–9,099,400)	< 0.001	< 0.001
DCP, mAU/ml	60 (10.0–10,300)	60 (8.0–211,120)	92 (10–11,013)	0.046	0.002
Child–Pugh classification				0.007	0.004
A	139 (93.3)	399 (94.1)	213 (98.6)		
B	10 (6.7)	25 (5.9)	3 (1.4)		
Tumor factors					
Primary tumor				0.993	0.171
T1-2	138 (92.6)	404 (95.3)	200 (92.6)		
T3-4	11 (7.4)	20 (4.7)	16 (7.4)		
Tumor size, cm	3.0 (1.1–27)	2.8 (0.9–15.5)	4.0 (0.5–20.0)	< 0.001	< 0.001
Tumor number				0.929	0.137
Single	116 (77.9)	309 (72.9)	169 (78.2)		
Multiple	33 (22.1)	115 (27.1)	47 (21.8)		
Fc-inf	82 (55.0)	242 (57.1)	123 (56.9)	0.718	0.975
Portal vein invasion	53 (35.6)	124 (29.3)	66 (30.6)	0.316	0.732
Hepatic vein invasion	16 (10.7)	36 (8.5)	38 (17.6)	0.065	0.001
Bile duct invasion	3 (2.0)	7 (1.7)	3 (1.4)	0.647	0.799
Intrahepatic metastasis	16 (10.7)	42 (9.9)	27 (12.5)	0.606	0.322
Edmondson-Steiner grade				0.384	0.717
1 or 2	113 (75.8)	310 (73.1)	155 (71.8)		
3 or 4	36 (24.2)	114 (26.9)	61 (28.2)		
Fibrosis stage^a^				0.002	< 0.001
F0-3	87 (58.4)	246 (58.0)	159 (73.6)		
F4	62 (41.6)	178 (42.0)	57 (26.4)		
Surgical factors					
Operation time, min	344 (145–712)	340 (100–990)	383 (82–1045)	0.018	< 0.001
Blood loss, mL	420 (0–4770)	450 (0–3960)	450 (0–6600)	0.314	0.702
Intraoperative PRBC	10 (6.7)	36 (8.5)	24 (11.1)	0.148	0.288
Resected liver weight, g	110 (4–3620)	87 (4–1800)	162 (2–2270)	0.044	< 0.001
Procedure				0.226	< 0.001
Anatomical resection	83 (55.7)	195 (46.0)	134 (62.1)		
Non-anatomical resection	66 (44.3)	229 (54.0)	82 (37.9)		
Surgical margin, mm	3.0 (0.0–35.0)	2.0 (0.0–44.0)	2.5 (0.0–60.0)	0.629	0.509
Total bilirubin max, mg/dl	1.5 (0.7–5.3)	1.4 (0.5–18.4)	1.5 (0.6–36.6)	0.662	0.038
PHLF				0.160	< 0.001
Grade A	12 (7.9)	13 (8.6)	25 (16.6)		
Grade B	17 (11.3)	62 (41.1)	19 (12.6)		
Major complication^b^	23 (15.4)	95 (22.4)	45 (20.8)	0.189	0.648
Postoperative hospital stay, days	22 (5–117)	24 (5–111)	17 (4–107)	< 0.001	< 0.001
Mortality	0 (0.0)	3 (0.7)	1 (0.4)	0.999	0.998

Data are presented as n (%) or median (range)

*HBV-HCC* hepatitis B virus-related hepatocellular carcinoma (hepatitis B surface antigen-positive), *HCV-HCC* hepatitis C virus-related hepatocellular carcinoma (hepatitis C antibody-positive), non-B non-C-HCC non-HBV non-HCV hepatocellular carcinoma, *BMI* body mass index, *HT* hypertension, *DM* diabetes mellitus, *Alb* albumin, *AST* aspartate aminotransferase, *ALT* alanine aminotransferase, *T.bil* total bilirubin, *Cre* creatinine, *Fib 4* fibrosis-4, *ICGR15* indocyanine green retention rate at 15 min, *AFP* α-fetoprotein, *DCP* des-gamma-carboxy prothrombin, *Fc-inf* frequency of tumor invasion to capsular formation, *PRBC* packed red blood cells, *PHLF* post-hepatectomy liver failure

^a^ According to the Shin-Inuyama classification

^b^ Major complications refer to grade III or IV events according to the Clavien–Dindo classification

With respect to host-related factors, the age of patients with non-B non-C-HCC was significantly higher than that of patients with HBV-HCC and HCV-HCC (*P* < 0.001 and *P* = 0.032, respectively). The incidence of patients with hypertension (HT), diabetes mellitus (DM), and excessive alcohol consumption was significantly higher among those with non-B non-C-HCC (49.1%, 47.2%, and 62.0%, respectively) than among those with HBV-HCC (22.7%, 11.4%, and 45.6%, respectively) and HCV-HCC (36.3%, 20.8%, and 38.7%, respectively). The blood platelet count and alanine aminotransferase concentration were significantly higher in patients with non-B non-C-HCC than in those with HBV-HCC and HCV-HCC, which was consistent with the lower prevalence of cirrhosis in patients with non-B non-C-HCC. With respect to tumor markers, the AFP and DCP concentrations were significantly higher in patients with non-B non-C-HCC than in patients with HBV-HCC and HCV-HCC, and the tumor size was greater in patients with non-B non-C-HCC than in patients with HBV-HCC and HCV-HCC. Although the operation time was longer and the resected liver weight was greater in patients with non-B non-C-HCC than in patients with HBV-HCC and HCV-HCC, the surgical margin and major complications (grade III or IV events according to the Clavien–Dindo classification) were comparable among the three groups.

### Survival after hepatic resection for HCC

**Fig. 2:**
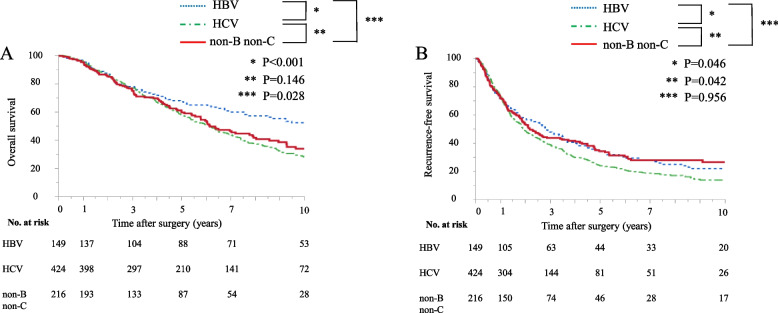
Kaplan–Meier survival analysis according to background of hepatocellular carcinoma. **a** Overall survival. **b** Recurrence-free survival. HBV, hepatitis B virus; HCV, hepatitis C virus; non-B non-C, non-HBV, non-HCV

The Kaplan–Meier survival curves for OS and RFS subdivided by background liver disease (HBV, HCV, and NBNC) are shown in Fig. 2. OS in patients with HCV-HCC and non-B non-C-HCC was significantly worse than that in patients with HBV-HCC, while it was comparable between patients with HCV-HCC and non-B non-C-HCC [5-year OS: 67.1% (HBV) vs. 57.9% (HCV) vs. 60.9% (NBNC), respectively; *P* < 0.001 (HBV vs. HCV), *P* = 0.146 (HCV vs. NBNC), *P* = 0.028 (HBV vs. NBNC)]. RFS in patients with HCV-HCC was significantly worse than that in patients with HBV-HCC and non-B non-C-HCC [5-year RFS: 34.1% (HBV) vs. 24.7% (HCV) vs. 34.2% (NBNC), respectively; *P* = 0.046 (HBV vs. HCV), *P* = 0.042 (HCV vs. NBNC), *P* = 0.956 (HBV vs. NBNC)].

### Prognostic factors for OS and RFS in patients with non-B non-C-HCC

The results of the multivariate analysis using the Cox proportional hazard model for predictors of OS in patients with non-B non-C-HCC are shown in Table 2. In the univariate analyses, 10 factors were found to be significant predictors. The multivariate analysis revealed that the independent poor prognostic factors were a DCP concentration of > 40 mIU/mL (HR: 1.64, 95% CI: 1.05–2.57, *P* = 0.029), Edmondson–Steiner grade 3 or 4 (HR: 1.73, 95% CI: 1.03–2.62, *P* = 0.035), operation time of > 480 min (HR: 2.15, 95% CI: 1.39–3.32, *P* < 0.001), non-anatomical resection (HR: 1.79, 95% CI: 1.16–2.75, *P =* 0.009), and major complications (HR: 1.64, 95% CI: 1.03–2.61, *P* = 0.038). In terms of RFS, 13 factors were found to be significant predictors of recurrence. The multivariate analysis revealed that the independent poor prognostic factors were an AFP concentration of > 100 ng/mL (HR: 1.75, 95% CI: 1.19–2.56, *P* = 0.004), multiple tumors (HR: 2.26, 95% CI: 1.32–3.88, *P* = 0.003), and an operation time of > 480 min (HR: 1.64, 95% CI: 1.08–2.49, *P* = 0.020) (Table 3).

**Table 2:** Prognostic factors for overall survival in patients with non-B non-C hepatocellular carcinoma

Variable	Univariate analysis			Multivariate analysis		
	HR	95% CI	*P* value	HR	95% CI	*P* value
Age > 70 years	1.01	0.69–1.47	0.961	-		
Gender (male)	1.27	0.76–2.13	0.370	-		
BMI > 22 kg/m^2^	0.71	0.48–1.06	0.094	-		
HT (yes)	1.02	0.59–1.74	0.951	-		
DM (yes)	1.23	0.85–1.80	0.271	-		
Dyslipidemia (yes)	0.72	0.17–2.96	0.645	-		
Heavy drinking (yes)	0.87	0.59–1.27	0.469	-		
Smoking (yes)	1.23	0.83–1.83	0.309	-		
Serum ALT > 40 IU/L	1.34	0.92–1.96	0.127	-		
Fib 4 score > 2.67	1.31	0.90–1.91	0.156	-		
Platelet count < 8.0 × 10^4^/ml	1.16	0.58–2.29	0.677	-		
ICGR15 > 10%	1.55	0.99–2.40	0.053	-		
AFP > 100 ng/ml	1.50	1.03–2.18	0.036	1.14	0.76–1.72	0.516
DCP > 40 mIU/ml	1.69	1.10–2.60	0.016	1.64	1.05–2.57	0.029
Primary tumor 3–4 (vs. 1–2)	1.40	0.70–2.77	0.339	-		
Tumor number (multiple)	1.82	1.19–2.80	0.006	1.27	0.77–2.09	0.342
Tumor size > 5 cm	1.20	0.81–1.76	0.361	-		
Fc-inf (yes)	1.39	0.92–2.08	0.115	-		
Portal vein invasion (yes)	1.89	1.01–3.55	0.047	1.47	0.72–3.00	0.284
Hepatic vein invasion (yes)	1.50	0.65–3.42	0.339	-		
IM (yes)	2.59	1.59–4.22	< 0.001	1.66	0.90–3.05	0.103
Edmondson-Steiner grade 3 or 4 (vs. 1 or 2)	1.73	1.13–2.63	0.011	1.65	1.03–2.62	0.035
Fibrosis stage 4 (vs. 0–3) ^a^	1.19	0.79–1.79	0.411	-		
Operation time > 480 min	1.94	1.30–2.92	0.001	2.15	1.39–3.32	< 0.001
Blood loss > 500 ml	1.30	0.89–1.89	0.172	-		
Inflow occlusion time > 60 min	0.97	0.67–1.42	0.889	-		
Non-anatomical resection	1.77	1.44–2.95	0.024	1.79	1.16–2.75	0.009
Surgical margin < 1 mm	1.71	1.12–2.61	0.013	1.05	0.63–1.76	0.844
Major complication ^b^	1.73	1.11–2.69	0.015	1.64	1.03–2.61	0.038

*HR* hazard ratio, *CI* confidence interval, *BMI* body mass index, *HT* hypertension, *DM* diabetes mellitus; non-B non-C, non-HBV, non-HCV; *ALT* alanine aminotransferase, *Fib 4* fibrosis-4, *ICGR15* indocyanine green retention rate at 15 min, *AFP* α-fetoprotein, *DCP* des-gamma-carboxy prothrombin, *Fc-inf* frequency of tumor invasion to capsular formation, *IM* intrahepatic metastasis, *PRBC* packed red blood cells

^a^ According to the Shin-Inuyama classification

^b^ Major complications refer to grade III or IV events according to the Clavien–Dindo classification

**Table 3:** Prognostic factors for recurrence-free survival in patients with non-B non-C hepatocellular carcinoma

Variable	Univariate analysis			Multivariate analysis		
	HR	95% CI	*P* value	HR	95% CI	*P* value
Age > 70 years	1.13	0.80–1.59	0.491	-		
Gender (male)	1.27	0.80–2.00	0.311	-		
BMI > 22 kg/m^2^	0.99	0.68–1.44	0.955	-		
HT (yes)	1.23	0.76–2.00	1.23	-		
DM (yes)	1.20	0.86–1.69	0.289	-		
Dyslipidemia (yes)	1.00	0.31–3.20	0.995	-		
Heavy drinking (yes)	0.97	0.69–1.37	0.869	-		
Smoking (yes)	1.00	0.72–1.42	0.986	-		
Serum ALT > 40 IU/L	1.79	1.27–2.53	< 0.001	1.45	0.99–2.11	0.053
Fib 4 score > 2.67	1.28	1.91–1.80	0.150	-		
Platelet count < 8.0 × 10^4^/ml	0.94	0.49–1.80	0.853	-		
ICGR15 > 10%	1.50	1.03–2.19	0.037	1.30	0.87–1.97	0.204
AFP > 100 ng/ml	2.14	1.52–3.02	< 0.001	1.75	1.19–2.56	0.004
DCP > 40 mIU/ml	1.69	1.16–2.46	0.007	1.35	0.89–2.06	0.158
Primary tumor 3–4 (vs. 1–2)	2.88	1.58–4.96	< 0.001	0.85	0.36–2.00	0.711
Tumor number (multiple)	2.75	1.87–4.04	< 0.001	2.26	1.32–3.88	0.003
Tumor size > 5 cm	1.85	1.31–2.61	< 0.001	1.51	0.98–2.32	0.059
Fc-inf (yes)	1.39	0.96–2.02	0.085	-		
Portal vein invasion (yes)	3.20	1.80–5.70	< 0.001	1.56	0.66–3.69	0.308
Hepatic vein invasion (yes)	4.23	2.26–7.90	< 0.001	1.58	0.70–3.52	0.266
IM (yes)	4.07	2.54–6.52	< 0.001	1.45	0.72–2.90	0.298
Edmondson-Steiner grade 3 or 4 (vs. 1 or 2)	1.44	0.98–2.10	0.060	-		
Fibrosis stage 4 (vs. 0–3) ^a^	1.38	0.96–1.99	0.080	-		
Operation time > 480 min	1.55	1.07–2.26	0.022	1.64	1.08–2.49	0.020
Blood loss > 500 ml	1.17	0.83–1.64	0.370	-		
Inflow occlusion time > 60 min	1.21	0.86–1.70	0.284	-		
Non-anatomical resection	0.84	0.59–1.01	0.331	-		
Surgical margin < 1 mm	1.49	1.01–2.21	0.046	0.99	0.63–1.56	0.969
Major complication ^b^	1.78	1.19–2.63	0.005	1.46	0.94–2.26	0.091

*HR* hazard ratio, *CI* confidence interval, *BMI* body mass index, *HT* hypertension, *DM* diabetes mellitus; non-B non-C, non-HBV, non-HCV; ALT, alanine aminotransferase; Fib 4, fibrosis-4; ICGR15, indocyanine green retention rate at 15 min; AFP, α-fetoprotein; DCP, des-gamma-carboxy prothrombin; Fc-inf, frequency of tumor invasion to capsular formation; IM, intrahepatic metastasis, PRBC, packed red blood cells

^a^ According to the Shin-Inuyama classification

^b^ Major complications refer to grade III or IV events according to the Clavien–Dindo classification

### Comparisons of OS and RFS among the three time periods

**Fig. 3:**
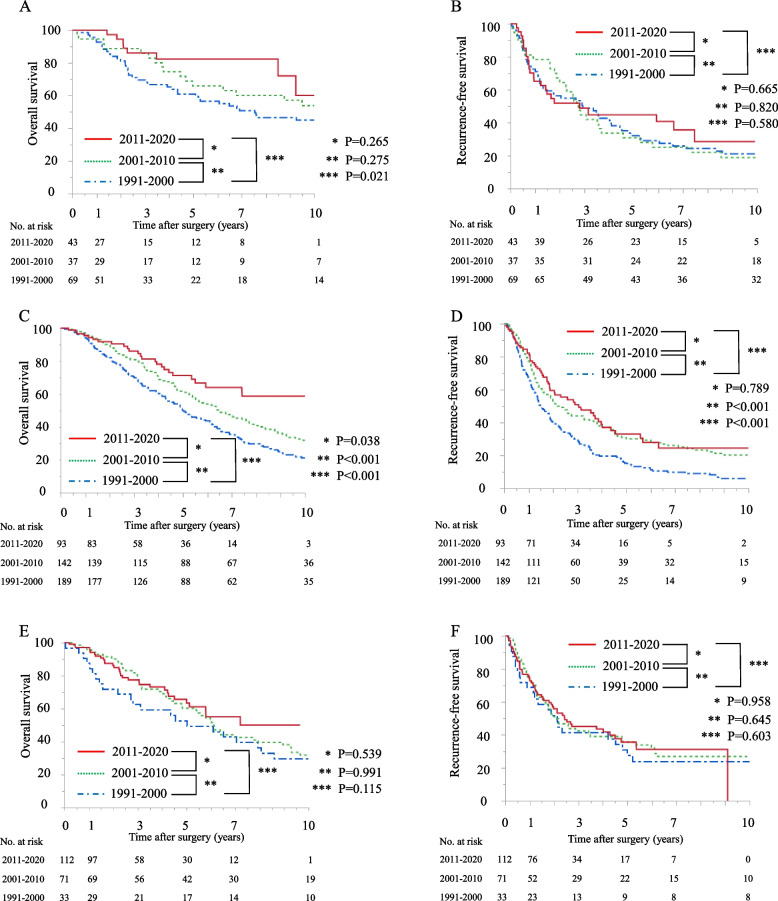
Kaplan–Meier survival analysis subdivided into three time periods. **a** OS in patients with HBV-HCC. **b** RFS in patients with HBV-HCC. **c** OS in patients with HCV-HCC. **d** RFS in patients with HCV-HCC. **e** OS in patients with non-B non-C-HCC. **f** RFS in patients with non-B non-C-HCC. OS, overall survival; RFS, recurrence-free survival; HBV, hepatitis B virus; HCV, hepatitis C virus; non-B non-C, non-HBV, non-HCV; HCC, hepatocellular carcinoma

The observational periods were divided into three groups as follows: period 1, 1990–2000; period 2, 2001–2010; and period 3, 2011–2020. In each of these periods, surgical treatment outcomes were compared according to the patients’ background factors. In patients with HBV-HCC, although RFS was comparable among the three periods, OS was significantly better in period 3 than in period 1 (5-year OS: 82.3% vs. 60.9%, *P* = 0.021) (Fig. 3a, b). In patients with HCV-HCC, both OS and RFS were significantly better in period 3 than in period 1 (5-year OS: 71.3% vs. 50.0%, *P* < 0.001; 5-year RFS: 33.1% vs. 15.2%, *P* < 0.001) (Fig. 3c, d). However, in patients with non-B non-C-HCC, both OS and RFS were comparable among the three periods (Fig. 3e, f).

### Comparisons of clinical characteristics and surgical outcomes among the three time periods in patients with non-B non-C-HCC

The differences in clinical characteristics, pathological findings, and surgical short-term outcomes among the three periods are summarized in Table 4. Age at surgery was significantly lower in period 1 than in period 2 (*P* < 0.001). The incidence of patients with HT, DM, dyslipidemia was significantly higher in period 3 than in period 1 (HT: 29.5% vs. 12.1%, *P* < 0.001; DM: 55.4% vs. 30.3%, *P* = 0.017; dyslipidemia: 20.5% vs. 12.1%, *P* < 0.001). Liver function, including the ICGR15 and liver fibrosis, was significantly better in period 3 than in period 1, whereas the AFP and DCP concentrations were significantly higher in period 3. With respect to surgical factors, the surgical outcomes (including the operation time, blood loss, post-hepatectomy liver failure rate, and postoperative hospital stay) were significantly better in period 3 than in period 1.

**Table 4:** Comparisons of clinical characteristics and surgical outcomes according to the three time periods in patients with non-B non-C –HCC

Variable	Period 1 1990–2000 (*n* = 33)	Period 2 2001–2010 (*n* = 71)	Period 3 2011–2020 (*n* = 112)	*P* value	
				Period 1 vs. Period 3	Period 2 vs. Period 3
Host-related factors					
Age, years	63 (33–80)	71 (41–88)	72 (41–89)	< 0.001	0.702
Sex, male/female	26/7	60/11	87/25	0.892	0.252
BMI, kg/m^2^	23 (18–30)	23 (14–36)	23 (13–45)	0.580	0.807
HT	4 (12.1)	19 (26.8)	33 (29.5)	< 0.001	0.088
DM	10 (30.3)	30 (42.3)	62 (55.4)	0.017	0.096
Dyslipidemia	4 (12.1)	7 (9.9)	23 (20.5)	< 0.001	< 0.001
Heavy drinking	19 (57.6)	40 (56.3)	75 (67.0)	0.407	0.160
Smoking	22 (66.7)	46 (64.8)	71 (63.4)	0.712	0.881
Serum Alb, g/dl	4.0 (3.3–4.7)	3.8 (3.0–4.5)	4.0 (2.8–5.2)	0.723	0.055
Serum AST, IU/L	38 (10–139)	33 (13–89)	33 (12–142)	0.096	0.856
Serum ALT, IU/L	33 (8–95)	34 (9–118)	35 (8–215)	0.445	0.635
Serum T.bil, mg/dl	0.8 (0.4–1.9)	0.7 (0.4–2.1)	0.8 (0.3–2.8)	0.823	0.056
Serum Cre, mg/dl	0.7 (0.0–11.6)	0.8 (0.4–2.3)	0.8 (0.4–11.5)	0.051	0.068
Prothrombin time, %	87 (50–119)	94 (32–130)	86 (61–119)	0.453	< 0.001
Platelet count, 10^4^/ml	14.9 (4.4–34.7)	16.5 (7.7–34.2)	16.3 (4.1–41.4)	0.734	0.114
Fib 4 index	4.0 (0.4–14.5)	2.4 (1.2–5.1)	2.6 (0.6–10.7)	0.224	0.352
ICGR15, %	13 (4–52)	14 (4–89)	10 (3–46)	0.006	0.006
AFP, ng/ml	12 (26–909,940)	88 (0–1,868,000)	66 (10–717,890)	0.001	0.893
DCP, mAU/ml	32 (10–34,700)	121 (10–255,600)	86 (12–102,283)	< 0.001	0.419
Child–Pugh classification				0.308	0.843
A	33 (100.0)	70 (98.6)	110 (98.2)		
B	0 (0.0)	1 (1.4)	2 (1.8)		
Tumor factors					
Primary tumor				0.117	0.911
T1-2	33 (100.0)	65 (91.6)	102 (91.1)		
T3-4	0 (0.0)	6 (8.4)	10 (8.9)		
Tumor size, cm	4.0 (1.1–20)	4.4 (1.3–15.0)	3.7 (0.5–16.5)	0.246	0.056
Tumor number				0.224	0.910
Single	29 (87.9)	54 (76.1)	86 (76.8)		
Multiple	4 (12.1)	17 (23.9)	26 (23.2)		
Fc-inf	23 (69.7)	13 (18.3)	17 (15.2)	< 0.001	0.683
Portal vein invasion	6 (18.2)	6 (8.5)	3 (2.7)	0.005	0.092
Hepatic vein invasion	4 (12.1)	4 (5.6)	4 (3.6)	0.079	0.713
Bile duct invasion	1 (3.0)	1 (1.4)	0 (0.0)	0.228	0.388
Intrahepatic metastasis	11 (33.3)	8 (11.3)	8 (7.1)	< 0.001	0.422
Edmondson-Steiner grade				0.671	0.094
1 or 2	24 (72.7)	56 (78.9)	75 (67.0)		
3 or 4	9 (27.3)	15 (21.1)	37 (23.0)		
Fibrosis stage ^a^				0.003	0.142
F0-3	15 (45.5)	60 (84.5)	84 (75.0)		
F4	18 (54.6)	11 (15.5)	28 (25.0)		
Surgical factors					
Operation time, min	415 (247–1045)	397 (106–846)	365 (82–798)	0.023	0.241
Blood loss, mL	868 (200–3600)	450 (19–5500)	400 (0–6600)	< 0.001	0.062
Intraoperative PRBC	6 (18.2)	7 (9.9)	11 (9.8)	0.220	0.993
Resected liver weight, g	200 (15–2270)	164 (14–1491)	152 (2–1655)	0.382	0.500
Procedure				0.418	0.999
Anatomical resection	18 (54.5)	45 (63.4)	71 (63.4)		
Non- anatomical resection	15 (45.5)	26 (36.6)	41 (36.6)		
Surgical margin, mm	1.0 (0.0–18.0)	3.0 (0.0–25.0)	3.0 (0.0–60.0)	0.002	0.317
Total bilirubin max, mg/dl	1.6 (0.6–12.9)	1.5 (0.6–4.1)	1.5 (0.6–36.6)	0.825	0.670
PHLF				< 0.001	0.098
Grade A	0 (0.0)	4 (5.6)	21 (18.8)		
Grade B	9 (27.3)	5 (7.0)	5 (4.5)		
Major complication ^b^	10 (30.3)	13 (18.1)	22 (19.6)	0.233	0.850
Postoperative hospital stay, days	24 (14–107)	21 (9–105)	12 (4–76)	< 0.001	< 0.001
Mortality	0 (0.0)	0 (0.0)	1 (0.9)	0.999	0.999

Data are presented as n (%) or median (range)

non-B non-C -HCC, non-HBV, non-HCV hepatocellular carcinoma; *BMI* body mass index, *HT* hypertension, *DM* diabetes mellitus, *Alb* albumin, *AST* aspartate aminotransferase, *ALT* alanine aminotransferase, *T.bil* total bilirubin, *Cre* creatinine, *Fib 4* fibrosis-4, *ICGR15* indocyanine green retention rate at 15 min, *AFP* α-fetoprotein, *DCP* des-gamma-carboxy prothrombin, *Fc-inf* frequency of tumor invasion to capsular formation, *PRBC* packed red blood cells, *PHLF* post-hepatectomy liver failure

^a^ According to the Shin-Inuyama classification

^b^ Major complications refer to grade III or IV events according to the Clavien–Dindo classification

## Discussion

The aim of this study was to characterize the clinical features and survival outcomes after surgical treatment of HCC among patients with background HBV, HCV, and NBNC and to clarify the surgical outcomes of the increasing number of patients with non-B non-C-HCC. Almost all previously reported studies concluded that OS or RFS after surgery for non-B non-C-HCC was significantly better than that after surgery for HBV-HCC or HCV-HCC, or the survival rates were the same. Similar results were obtained in the present study. However, in the study by Hsu et al. [9], patients with non-B non-C-HCC had a higher incidence of HT, DM, dyslipidemia, excessive alcohol consumption, and a current smoking habit than their counterparts. Additionally, although patients with non-B non-C-HCC had a lower fibrosis stage and better liver function (as indicated by measures such as the ICGR15) than their counterparts, they also had more advanced HCC, a greater tumor size or more severe vascular invasion, and worse OS and RFS [9]. Indeed, more advanced primary tumor stages were observed in patients with non-B non-C-HCC in this study. This result is consistent with past reports [3, 5, 8, 21–24]. This may be due to the lack of systematic surveillance of potential candidates for non-B non-C-HCC resection compared with HBV-HCC and HCV-HCC resection.

The prevalence of patients with non-B non-C-HCC has been increasing each year, and it has reached 10% to 20% in Asia [25]. In our institution, the number of patients with HBV-HCC and HCV-HCC has been decreasing probably due to advances in medical treatments such as nucleoside or nucleotide analogues and interferons, whereas the number of patients with non-B non-C-HCC has been dramatically increasing in recent years. The most likely reason behind these changes may be the increasing prevalence of metabolic syndrome [26]. Many studies have revealed an important association between the development of non-B non-C-HCC and metabolic disorder [3, 9, 10, 26]. Lifestyle-related diseases (e.g., HT, DM, and dyslipidemia), excessive alcohol consumption, and current smoking may also be associated with the development of non-B non-C-HCC; however, the pathogenic mechanisms underlying the development of non-B non-C-HCC remain elusive. At this stage, we are still in need of analysis in a lot of studies for non-B non-C-HCC; the same can be said for surgical therapy, which is at the core of treatment for HCC.

As indicated in this study, the postoperative outcomes (particularly OS) for both patients with HBV-HCC and patients with HCV-HCC have improved during the past 30 years. However, patients with non-B non-C-HCC are the most concerning because their prognosis has not substantially improved. First, our study showed that the incidence of HT, DM, and dyslipidemia in patients with non-B non-C-HCC significantly increased. In addition, tumor markers were significantly elevated despite good liver function and controlled fibrosis. Although this was a concern because of the selection bias (i.e., patients with poor liver function might not have undergone surgical resection), so-called lifestyle-related diseases were almost certainly deeply involved in the pathogenesis of non-B non-C-HCC. The tumor marker concentrations were increased at the time of surgery, and the lack of improvement in the long-term prognosis despite improved surgical outcomes indicates that early surgery might be the key to an improved prognosis. Therefore, the problem of early detection of non-B non-C-HCC should be solved. Second, in patients with viral hepatitis, it is possible to reduce the risk of carcinogenesis (reduce the risk of recurrence and improve liver function deterioration) by treating the underlying liver disease (the hepatitis itself), and advances in antiviral therapy are expected to improve the long-term prognosis. In patients with non-B non-C-HCC, however, no breakthrough treatment to reduce the risk of carcinogenesis has been established, and this might be the cause of the disease.

Nevertheless, the etiology of carcinogenesis or the tumor microenvironment in non-B non-C-HCC is naturally different from that of HBV-HCC and HCV-HCC. The immune mechanisms of NASH-HCC have recently been elucidated [27, 28]. Studies revealed that patients with NASH-driven HCC who received treatment with anti-programmed death receptor-1 or anti-programmed cell death ligand 1 showed lower OS than patients with other etiologies. non-B non-C-HCC, particularly NASH-HCC, might be less responsive to immunotherapy than its counterparts. Thus, differences and changes in the cancer immune-microenvironment and molecular oncological differences due to background liver disease are likely to be involved in cancer development and the risk of multicentric carcinogenesis. This may have profound implications for inoperable non-B non-C-HCC and the choice of neoadjuvant chemotherapy for conversion. Although the clinical characteristics, carcinogenic mechanisms, and optimal treatment strategy of non-B non-C-HCC need to be established as soon as possible, it is very important to research the molecular oncology of how each factor interacts with the others.

The present study had some limitations. First, the main limitation is that the observational duration was relatively long. Second, it was a retrospective study that was conducted at a single institution and may therefore have been subject to selection bias. Third, the treatment periods were divided into three groups, and the results might have differed if different cut-off periods had been selected. Despite these drawbacks, our results highlight the clinical features and outcomes of patients with non-B non-C-HCC after hepatectomy, which may help surgeons to select the most appropriate treatments in these patients.

In conclusion, patients with non-B non-C-HCC have a high prevalence of lifestyle-related disease or excessive alcohol consumption and current smoking, and their postoperative prognosis is comparable to that of patients with HBV-HCC and HCV-HCC regardless of tumor progression at the time of surgery. Therefore, further systematic follow-up is needed for patients with non-alcoholic fatty liver disease or NASH, and the early establishment of drugs for preventing HCC development or recurrence from NBNC-hepatitis is desired.

## Data Availability

Data cannot be shared publicly because of the necessity to protect personal information. However, they are available from the Shinshu Institutional Data Access / Ethics Committee (contact via shinhp@shinshu-u.ac.jp) for researchers who meet the criteria for access to confidential data. The data underlying the results presented in the study are available from Shinshu University (shinhp@shinshu-u.ac.jp).
